# Effect of autolysis on the specificity of bovine spongiform encephalopathy rapid tests

**DOI:** 10.1186/1756-0500-3-193

**Published:** 2010-07-14

**Authors:** Daniela Meloni, Katia Varello, Marzia Pezzolato, Elsa Manzardo, Maria C Cavarretta, Francesco Ingravalle, Maria Caramelli, Elena Bozzetta

**Affiliations:** 1 National Reference Laboratory for Animal Encephalopathies, Istituto Zooprofilattico Sperimentale del Piemonte Liguria e Valle d'Aosta, Turin, Italy

## Abstract

**Background:**

Routine rapid testing for Bovine Spongiform Encephalopathy (BSE) has highlighted some problems with BSE rapid test performance, the most significant being the number of initially reactive samples and the false positive results on autolyzed tissue. This point is important for BSE active surveillance in risk populations, because tissue autolysis is often unavoidable in routine cases. A robust test suitable for use on field material is therefore needed. To date, very limited information regarding the effect of autolysis on the robustness of rapid tests has been documented; therefore, the National Reference Centre for Animal Encephalopathies (CEA) rapid test laboratory selected 450 autolyzed and negative brain stem samples from fallen stock bovines older than 24 months to assess the specificity of four tests approved for BSE active surveillance: Biorad TeSeE, Enfer TSE version 2.0, Prionics^® ^Check LIA, and IDEXX Herd Check BSE Antigen Kit EIA. The samples were graded according to the degree of autolysis and then dissected into five portions, four of which randomly assigned to processing by rapid tests and one to be available for confirmatory Western blot analysis.

**Findings:**

The specificity of the four systems was 100% for all three grades of autolysis, while the percentage of initially reactive results was 0.00 (95%CI 0.00-0.82), 0.22 (95%CI 0.006-1.23), 0.44 (95%CI 0.05-1.60), and 0.89 (95%CI 0.24-2.26) for the Biorad TeSeE, the Prionics^® ^Check LIA, the IDEXX Herd Check BSE and the Enfer TSE tests, respectively. No association with the degree of autolysis could be drawn.

**Conclusions:**

The present study demonstrates that the four rapid tests can be considered well-running diagnostic tools regardless of tissue quality; nevertheless, the number of initial reactive samples reported for some systems must not be underestimated in routine testing.

Furthermore the compliance with the reported performance can be guaranteed only when an ongoing high careful batch quality control system is in place.

## Background

Bovine spongiform encephalopathy (BSE), a fatal infectious neurodegenerative disease of cattle, is characterized by the concentration of an anomalous isoform of the natural prion protein (PrP^c^), denominated PrP^res^, in the central nervous system (CNS) [1,2]. PrP^res ^differs from PrP^c ^in its high insolubility and partial protease resistance: these characteristics are exploited by the majority of the methods currently used for BSE diagnosis. Evidence for the origin of variant Creutzfeldt-Jakob Disease (vCJD) from BSE [3,4] led the European Commission (EC) to strengthen control programs, introducing active surveillance by means of rapid tests to monitor the ruminant infection [5]. Since 1999 the EC has assessed 19 rapid tests, 9 of which were approved for survey including a new version of the Enfer TSE test for BSE diagnosis [6].

In 1999 the EC carried out the first scientific evaluation of four new rapid *post mortem *BSE tests in which diagnostic accuracy and analytic sensitivity on brain tissue from clinically affected bovines was assessed [7]. Subsequent EU validation exercises extended assessment parameters to include test robustness on autolyzed material as a fundamental criterion, simulating routine field diagnosis [8-10].

Active surveillance samples come primarily from slaughtered animals; for this target category the caudal brainstem is collected immediately after the animal's death and sent to the laboratory. But a fairly high percentage of samples (13.5% in 2007 in EU Member States [11]) come from fallen stock cattle; in Italy, some 90% of samples arrive at the laboratory in condition of autolysis, owing to the time between the animal's death and collectionof the sample.

Previous studies investigated the effect of autolytic changes on the detection of Scrapie-associated fibrils (SAF) [12,13] and then on PrP^res ^immunodetection by immunoblotting and immunohistochemistry [14-17]. The ability of some rapid test systems to correctly classify BSE autolyzed positive samples was also ascertained [18]. The outcome of those studies was an undiminished sensitivity for PrP^res ^detection for all diagnostic methods evaluated, even when applied to severely autolyzed tissues.

To date, there is a little reliable information [19] about the effect autolysis can have on rapid test performance for large-scale throughput samples in terms of number of initially reactive samples and false positive results. To fill this gap, we wanted to determine whether natural autolysis affected the performance of four rapid tests commonly used in the EU for mandatory surveillance of BSE (Prionics^® ^Check-LIA Test, Enfer TSE Kit version 2.0, Bio-Rad TeSeE Test, IDEXX HerdChek BSE Antigen Test Kit EIA) on negative and autolyzed field samples.

## Methods

### Samples

A total of 450 autolyzed bovine brain stem samples (*medulla oblongata *at the level of the obex) from fallen stock animals older than 24 months, sent to the Rapid Test Laboratory of the National Reference Centre for TSE (CEA - National Reference Laboratory [NRL]) for BSE monitoring, were collected from June to September 2008 and assigned a progressive number.

### Protocol

Samples coming to the laboratory were graded according to the degree of autolysis as follows:

■ Grade 1: reduced consistence, obex easily recognizable, absence of abnormal colours;

■ Grade 2: reduced consistence, obex not always recognizable, presence of abnormal colours;

■ Grade 3: liquid state, obex not recognizable, presence of abnormal colours.

Up to 150 samples were collected for each grade of autolysis.

Three grams of recognizable obex tissue were dissected and carefully minced. Approximately 0.6 g of tissue were randomly assigned to each rapid test:

■ Prionics^® ^Check-LIA Test is a microplate-based immunoassay (ELISA) that uses monoclonal antibodies to detect proteinase K resistant PrP^res ^(Test A);

■ Enfer TSE Kit version 2.0 automated sample preparation is a chemiluminescent ELISA test involving an extraction procedure and an ELISA technique that uses an enhanced chemiluminescent reagent (Test B);

■ Bio-Rad TeSeE Test uses a sandwich immunoassay technique to detect PrP^res ^following denaturation and concentration steps (Test C);

■ IDEXX HerdChek BSE Antigen Test Kit EIA is an immunoassay that uses a chemical polymer for selective PrP^res ^capture and a monoclonal detection antibody directed against the conserved regions of the PrP molecule (Test D);.

The fifth aliquot was stored to be available for confirmatory Western blot assay in case of positive results to the rapid tests.

For completely autolyzed samples in which the obex region was not identifiable, the aliquot was taken from the same tissue area.

The samples were analyzed according to CEA standard operating protocols based on the test manufacturer's instructions.

### Statistics

Percentage of initially reactive samples and specificity with relative confidence limits at 95% was calculated using STATA [20] software.

### Ethical approval

The samples were collected according to OIE Manual of Diagnostic Tests and Vaccines for Terrestrial Animals 2008 [21] standard operating protocols in the frame of Regulation (EC) No 999/2001 [5] dispositions.

### Results and discussion

Table 1 reports the number and the percentage of initially reactive samples by grade of autolysis, as a whole, and the specificity. Differences among classes of autolysis did not result in statistically significant.

**Table 1:** Number and percentage of initially reactive samples and specificity

Test	Grade 1		Grade 2		Grade 3		Total	Specificity
	**N**	**%**	**N**	**%**	**N**	**%**	**%**	**%**

BIORAD TeSeE	0	0.00%	0	0.00%	0	0.00%	0.00%	100%
		(95% CI 0.00-2.43)*		(95% CI 0.00-2.43)*		(95% CI 0.00-2.43)*	(95% CI 0.00-0.82)	(95% CI 99.18-100)*

ENFER TSE	2	1.33%	1	0.67%	1	0.67%	0.89%	100%
version 2.0		(95% CI 0.16%-4.73)		(95% CI 0.02-3.66)		(95% CI 0.02-3.66)	(95% CI 0.24-2.26)	(95% CI 99.18-100)*

Prionics Check LIA	1	0.67%	0	0.00%	0	0.00%	0.22%	100%
		(95% CI 0.02-3.66)		(95% CI 0.00-2.43)*		(95% CI 0.00-2.43)*	(95% CI 0.006-1.23)	(95% CI 99.18-100)*

IDEXX HerdChek BSE	0	0.00%	0	0.00%	2	1.33%	0.44%	100%
		(95% CI 0.00-2.43)*		(95% CI 0.00-2.43)*		(95% CI 0.16-4.73)	(95% CI 0.05-1.60)	(95% CI 99.18-100)*

(*) one-sided, 97.5% confidence interval

In rapid test validation studies, the objective of field trials is to demonstrate that the performance of a new diagnostic system is not inferior to those already approved for active surveillance. For this purpose, the diagnostic sensitivity and specificity of a new rapid test and its robustness are evaluated in field trials carried out by national reference laboratories (NRL) which run a high volume of samples of heterogeneous quality in parallel with the test in use. All diagnostic tests produce false positives and false negatives, but the best keep both rates (error probabilities) to nearly zero. In the recent BSE scenario, in which the epidemic is reported on the decline in most countries, with only two cases detected in Italy in 2009, the specificity of the systems in use is crucial: false reports of new cases unnecessarily alarm public opinion and even a few false positives can greatly overstep the true positives a test will detect [7].

Field trials provide test manufacturers with a better opportunity to acquire practical experience and to develop the user manual final version. That said, the real robustness of a test emerges only when it is applied during routine testing, in different climatic conditions and on poor quality samples. For this purpose, we tested only naturally autolyzed samples as documented by previous studies (Wear et al., 2005) to better mimic routine field conditions.

According to Italian NRL data, some laboratories regularly obtain a large number of initially reactive results from poor-quality samples. This is why verifying the robustness of rapid tests on autolyzed samples becomes relevant for BSE surveillance and also for correcting potential technical problems.

The meaning of surveillance measures could be affected if rapid test performance is influenced by the autolysis state *per se *or because of the loss of neuroanatomy of the target area. In this connection, previous studies [15,16] reported that while correct sampling is important, tissue quality appears to be a minor factor influencing false results of a test.

Here we show that all four rapid tests displayed 100% specificity even in the severely autolyzed samples. However, differences in test performance on initially reactive samples were noted, with test C resulting the best-performing system. For the other three tests, a direct link between the number of initially reactive routes and the degree of autolysis could not be highlighted either when initially reactives were taken as a whole or related to a specific test.

The test A underwent the field trial in 2002, but no initial reactivity on poor-quality samples was reported. Our results on this test are in accordance with those obtained in that frame, while a recent paper from Carra et all [19] on the evaluation of test A in routine monitoring activity reports a high prevalence of false positive results and frequencies of retested samples on fresh matrix and, with a higher percentage, on poor quality tissues. These data could be referred both to individual batch issues and/or local technical matters.

Nevertheless, it's worth to be considered that in the EFSA Scientific Opinion on Analytical sensitivity of approved TSE rapid tests of 2009 [22] a striking rate of initial reactive results (100%) achieved on reference negative tissue samples prepared both according to the manufacturer protocol, both with the Central Reference Laboratory (CRL) for TSE of Weybridge protocol (water:tissue 50:50), is reported for test A. No repeat testing was done on initial reactives in the context of this TSE analytical sensitivity study. Therefore, analytical sensitivity of test A has been recently re-assessed by CRL for TSEs [23] on different sets of reference positive and control negative dilutions prepared according to the manufacturer protocol without having initial reactive results on negative samples. Nevertheless the causes of previous initial reactives remain unidentified. Such situations could highlight a major problem in the quality control system put in place by the manufacturer, causing a failure in the performances of single batches released for routine activity, independently from the condition of sample under investigation. As a matter of fact test A is the only one among the approved BSE rapid test still not undergoing the official batch testing system, in place since 2008 under the responsibility of NRLs [24] before the admittance within the EU market.

No initially reactive samples for either the tests B and D were shown in 2005 field trial. Our results regarding initial reactive samples by these tests as a whole can be compared to those obtained in EU field trial. The high number of autolyzed samples tested in this study can partially explain the apparent differences from the EU evaluation data, in which the number of autolytic samples tested was limited (200). This means that, during the EU field trials, the confidence interval at 95% (95% CI) for the number of initially reactive samples relative to 200 poor-quality samples tested equals 0% - 1.83%. The percentage of initially reactive samples in a worst-case scenario of our study would be 0.89% (4/450 for test D), which is compatible with the 95% CI of the EU field trial. Furthermore, as already reported for test A, local technical issues related to temperature and humidity can not be ignored. Finally, test C was evaluated as "CEA" test in 1999, but no evaluation of robustness was provided.

## Conclusions

Initially reactive samples need to be retested, as expected with all test procedures, which leads to increased laboratory workload. Furthermore, samples producing false positive results at retesting can create false alarms and require time-consuming confirmatory testing. This, in turn, triggers a cascade of public health measures, including herd quarantine, animal transportation and slaughter prohibition and bans on commercialization of milk and by-products until the diagnostic result is confirmed by the NRL. In the meantime, an economically wasteful scare begins to spread across the market. To avert such situations, the screening purpose of rapid tests dictates that they perform equally well on autolyzed and good-quality samples alike.

In our study, the poor-quality tissues were still adequate for testing, highlighting that the robustness of the techniques are not compromised under adverse conditions of temperature and time between slaughter and testing. Nonetheless, the problem of initially reactive samples with regard to some diagnostic systems, must be strategically considered in laboratories with large workloads.

Other, it should be looked on that unresolved problems related to the quality control system applied during the manufacture of new test batches can severely affect the performances of rapid tests.

## Competing interests

The authors declare that they have no competing interests.

## Authors' contributions

All authors read and approved the final manuscript. DM have made substantial contribution to conception, drafting, revising, collecting data and analysis interpretation. KV have been involved in drafting the manuscript. MP have been involved in revising the manuscript. EM have made substantial contribution to the analytical examination. MCC have made substantial contribution to the analytical examination. FI have made substantial contribution to the statistical interpretation of the results. MC have given final approval of the version to be published. EB have made substantial contribution to conception, design, revising and analysis interpretation.

## Appendix

### Sources and manufacturers

A. Prionics^® ^Check LIA Test, Prionics AG, Wagistrasse 27a, CH-8952 Schlieren-Zurich, Switzerland.

B. Enfer TSE test, Enfer Scientific Limited Unit T, M7 Business Park Newhall, Naas, Co. Kildare, Ireland

C. Bio-Rad TeSeE, Bio-Rad, 3, Boulevard Raymond Poincaré, F-92430 Marnes la Coquette, France.

D. IDEXX Herd Check BSE Antigen Kit EIA, Idexx Laboratories Inc., Westbrook, ME, U.S.
